# Tannin-rich extracts from *Lannea stuhlmannii* and *Lannea humilis* (Anacardiaceae) exhibit hepatoprotective activities *in vivo via* enhancement of the anti-apoptotic protein Bcl-2

**DOI:** 10.1038/s41598-018-27452-8

**Published:** 2018-06-19

**Authors:** Mansour Sobeh, Mona F. Mahmoud, Rehab A. Hasan, Mohamed A. O. Abdelfattah, Omar M. Sabry, Mosad A. Ghareeb, Assem M. El-Shazly, Michael Wink

**Affiliations:** 1 Institute of Pharmacy and Molecular Biotechnology, Heidelberg University, Im Neuenheimer Feld 364, 69120 Heidelberg, Germany; 2 Department of Pharmacology and Toxicology, Faculty of Pharmacy, Zagazig University, Zagazig, 44519 Egypt; 3 Department of Histology, Faculty of Medicine for Girls, Al-Azhar University, Cairo, 11651 Egypt; 4 Department of Chemistry, American University of the Middle East, Egaila, 54200 Kuwait; 5 Department of Pharmacognosy, College of Pharmacy, Cairo University, Cairo, 11562 Egypt; 6 Medicinal Chemistry Department, Theodor Bilharz Research Institute, Kornish El-Nile, Warrak El-Hadar 12411, Imbaba, Giza, Egypt; 7 Department of Pharmacognosy, Faculty of Pharmacy, Zagazig University, Zagazig, 44519 Egypt

## Abstract

The potential hepatoprotective activities of two *Lannea* species were explored *in vivo*. Furthermore, the binding activities of their main polyphenols to the antiapoptotic protein Bcl-2 were investigated. Based on HPLC-MS/MS results, 22 secondary metabolites were characterized in *L*. *stuhlmannii* (mainly tannins), while 20 secondary metabolites (mainly sulphated tannins) were identified in *L*. *humilis*. Both extracts exhibited substantial antioxidant activities *in vitro* and counteracted D-galactosamine induced intoxication in rats *in vivo* and increased the total antioxidant capacity (TAC) of liver tissues. In addition to reducing the elevated levels of AST and total bilirubin, both extracts significantly attenuated the deleterious histopathologic changes in liver after D-galactosamine-intoxication. Also, both extracts protected hepatocytes from apoptotic cell death and increased the expression of the anti-apoptotic protein Bcl-2. The identified compounds from both extracts can bind to the Bcl-2: Bim (BH3) interface with an appreciable binding free energy. Hydrogen and ionic bonds and hydrophobic interactions with amino acid residues in the hydrophobic face of Bim (BH3) domain were discovered. To sum up, *L*. *humilis* and *L*. *stuhlmanni* exhibited promising hepatoprotective activities *in vivo* against D-GalN-induced liver injury and their hepatoprotection is due to the antioxidant and anti-apoptotic effects of tannins and proanthocyanidins.

## Introduction

Liver cirrhosis is considered a major health problem and a leading cause of death all over the world. This pathological condition is directly or indirectly connected to an overproduction of free radicals. Drug candidates able to combat free radicals and their negative impact on cells could serve as leads for treatment. Several studies have demonstrated that many plants produce secondary metabolites with antioxidant and proven powerful free radical scavenging activities. Such plants might have a potential for the treatment of liver diseases linked to oxidative stress and related conditions^1,2^.

The genus *Lannea* with 40 species is one of 69 genera (about 850 species) belonging to the Anacardiaceae. This genus, along with others such as *Pistacia*, *Blepharocarya*, *Campylopetalum*, and *Dobinea*, is widespread in the tropics^3^. Phytochemical investigations of several members of this genus revealed several new and rare natural products distributed over different phytochemical classes such as tannins, cyanidins, flavonoids, alkylphenols, and dihydroalkyhexenones^4,5^. For instance, a prenylated chromenflavanone was isolated from *L*. *acida* leaf extract^6^. Also, three dihydroalkylhexenones were discovered in *L*. *edulis* in a bio-guided fractionation^5^.

Several biological activities have been reported from the genus *Lannea*. In particular, a *L*. *coromandelica* leaf extract has shown anti-conceptive activities while its bark extract was found to have hypoglycemic activities, in addition to anti-inflammatory activities in rats^7,8^. Okoth *et al*. have reported antioxidant and antibacterial activities for *L*. *alata* root and the isolated compounds myricitrin, myricetin-3-*O*-*a*-arabinofuranoside, lupeol, and sitosterol^4^.

*Lannea stuhlmannii* and *L*. *humilis* are deciduous trees widely distributed in the tropics^3^. In Tanzania, *Lannea* species are being used traditionally for the treatment of cancer. Also, decoctions of *L*. *stuhlmannii* root are still in use against fever, asthma, stomachache, and dysentery while decoctions of the stembark are considered beneficial to counteract headache and stomach pain. The leaf extract is used to relieve abdominal pain and to hasten childbirth. Moreover, stembark extract has shown antibacterial activity^9^. In Namibia, *L*. *stuhlmannii* roots are used as ethnomedicine for the treatment of HIV/AIDS infections. In East Africa, the plant is employed traditionally in treatment of anaemia^10^.

The bark of *L*. *humilis* is used traditionally to treat fever, and diarrhea^11^. In Tanzania, a root decoction is drunk to treat anaemia, stomach pains, nausea and general body weakness^12^. *L*. *humilis* trees excrete a water-soluble gum rich in amino acids, galactose, and other polysaccharides^13,14^. The root of *L*. *stuhlmannii* and the bark from *L*. *humilis* demonstrated promising anti-trypanosomal and cytotoxic activities in a panel of 40 plants extracts^15^.

Phytochemical studies are missing for both species. However, a preliminary study by Chhabra *et al*. reported the presence of some alkaloids and flavonoids in *L*. *stuhlmannii* bark^16^. Herein we characterized the polyphenolic constituents of *L*. *stuhlmannii* and *L*. *humilis* bark extracts using HPLC-PDA-ESI-MS/MS. Moreover, *in vitro* antioxidant activities were studied and hepatoprotective activities were investigated in rats treated with the toxic D-galactosamine that causes liver damage. Molecular modelling was employed to find out if the main polyphenols of the plants bind to Bcl-2: Bim (BH3) interface that might be modulated by the drug.

## Materials and Methods

### Plant material

*Lannea humilis* (Oliv.) Engl. and *Lannea stuhlmannii* (Engl.) Engl. bark samples were collected from trees growing in Lupaga Site in Shinyanga, Tanzania and kept under accession number P7311 and P7317, respectively, at IPMB, Heidelberg University^15^. The bark samples were ground and extracted with 100% methanol at room temperature for three days (6 × 500 mL). The combined extracts were filtered and evaporated using a rotary evaporator at 40 °C. The residues were frozen at −70 °C, and then lyophilized to fine dried powder. The extraction yield was calculated based on initial dry weight and found to be 22% and 15% for *L*. *humilis* and *L*. *stuhlmannii* bark, respectively.

### HPLC-PDA-MS/MS

The extracts were analyzed utilizing a ThermoFinnigan LCQ-Duo ion trap mass spectrometer (ThermoElectron Corporation, Waltham, Ma, USA) with an ESI source (ThermoQuest Corporation, Austin, Tx, USA). A ThermoFinnigan HPLC system using a C18 reversed-phase column (Zorbax Eclipse XDB-C18, Rapid resolution, 4.6 × 150 mm, 3.5 µm, Agilent, Santa Clara, CA, USA) was used^17^. Water and acetonitrile (ACN) (Sigma-Aldrich GmbH, Germany) (0.1% formic acid each) were used as a mobile phase. At 0 min, ACN was 5% and then increased to 30% in 60 min at 1 mL/min with a 1:1 split before the ESI source. Autosampler surveyor ThermoQuest was utilized to inject the extracts and the system was controlled by Xcalibur software (Xcalibur^TM^ 2.0.7, Thermo Fischer Scientific, Waltham, Ma, USA). The MS operated in the negative mode as reported before^18^. The ions were detected in a full scan mode and mass range of 50–2000 *m/z*.

### Biological experiments

#### Antioxidant activities *in vitro*

The Folin-Ciocalteu method quantified the total phenolic content at the extracts as described before^19^. The 2,2′-diphenyl-1-picryl-hydrazyl-hydrate (DPPH) radical scavenging and ferric reducing antioxidant power (FRAP) assays were utilized to evaluate the antioxidant properties of the extracts and were carried out as described earlier^19^.

### Animals and experimental design

Male Wistar rats (8 weeks of age, Faculty of Veterinary Medicine, Zagazig, Egypt) were used. Rats were housed in a light-controlled room with a 12-h light/dark cycle and had access to food and water. Experimental protocols and animal care methods were approved by Ethical Committee of the Faculty of Pharmacy, Zagazig University for Animal Use (Zagazig, Egypt) and conducted following the guidelines of the US National Institutes of Health on animal care and use. After a 1-week acclimatization period, rats were divided into seven groups: (**1**) Control, (**2**) D-galactosamine (D-GalN) treated with vehicle (0.9% saline), (**3**) *Lannea humilis* (100 mg/kg, oral), (**4**) *L*. *humilis* (200 mg/kg, oral), (**5**) *Lannea stuhlmannii* (100 mg/kg, oral), (**6**) *L*. *stuhlmannii* (200 mg/kg, oral) and (**7**) silymarin (100 mg/kg, oral). Six animals were allocated to each group (total animals: n = 42). To induce acute liver failure, the rats were injected with D-GalN (800 mg/kg, i.p). The animals were treated with vehicle, extracts or silymarin for three consecutive days before the injection of D-GalN. Blood samples were drawn from the retro-orbital plexus of the eye 24 h after D-GalN injection under pentobarbital anesthesia (50 mg/kg, i.p.). The serum was separated by centrifugation at 3500 × *g* for 15 min at 4 °C. These serum samples were used for measurement of AST activity and total bilirubin level. The livers were excised and used for light microscopic observations, oxidative stress markers measurement and immunohistochemical staining of Bcl-2.

### Liver proteins

Serum aspartate aminotransferase (AST) activity was measured using a commercial assay kit, (Diamond diagnostic, Egypt). Total bilirubin levels were measured using commercially available analytical kit (BioMed Diagnostics, Egypt).

### Determination of oxidative stress markers

The generation of reactive oxygen species following D-GalN injection was detected in liver tissues using commercially available kits provided by Biodiagnostic (Giza, Egypt) to measure the lipid peroxidation product content, malondialdehyde (MDA).Measurement of Total antioxidant capacity (TAC) was performed by reacting with the endogenous antioxidants in the serum sample with certain amount of hydrogen peroxide (H_2_O_2_). The remaining H_2_O_2_ was measured by an enzymatic reaction with 3,5-dichloro-2-hydroxy benzene sulphonate producing a colored compound. The intensity of the resulting color was measured by spectrophotometer (JENWAY 6105 UV/V Spectrophotometer) at wavelength of 505 nm.

### Histology

Excised livers were processed for light microscopic examination, according to standard procedures. The livers were preserved in phosphate-buffered 10% formalin, after which the livers were cut into small pieces, embedded in paraffin wax, sectioned at 4 µm thickness, and stained with haematoxylin and eosin.

### Immunohistochemical determination of Bcl-2 (anti-apoptotic marker)

Liver sections were prepared by soaking them in xylene to remove paraffin and graded alcohol was used (70%, 90%, 100%). The activity of endogenous peroxidase was blocked by addition of 5% H_2_O_2_ in absolute methanol for 10 min at room temperature. Sections were then rinsed by phosphate buffered saline (PBS). Thereafter, sections were incubated with primary antibodies against Bcl-2. Protein expression was measured using Streptavidin biotin peroxidase kit. Tissues were stained with diaminobenzidine (DAB) as chromogen for Bcl-2 detection and then counterstained with haematoxylin^20^.

### Morphometric analysis

The number of Bcl-2 positive hepatocytes in five randomly selected high power microscopic fields were analysed using computerized image system (a Leica Qwin 500 image analyser connected to a Leica microscope). This number was expressed as cell number per µm².

### Statistical analysis

Values are expressed as the mean ± standard error of the mean. Data were processed by Graphpad prism version 5 (GraphPad Software, San Diego, CA, U.S.A.). For statistical analysis, we used unpaired Student’s *t*-tests for two-group comparisons and one-way analysis of variance (ANOVA) followed by Tukey *post hoc* tests for multiple comparisons. For all comparisons, differences were considered significant at *p* < 0.05.

## Results and Discussion

**Figure 1:**
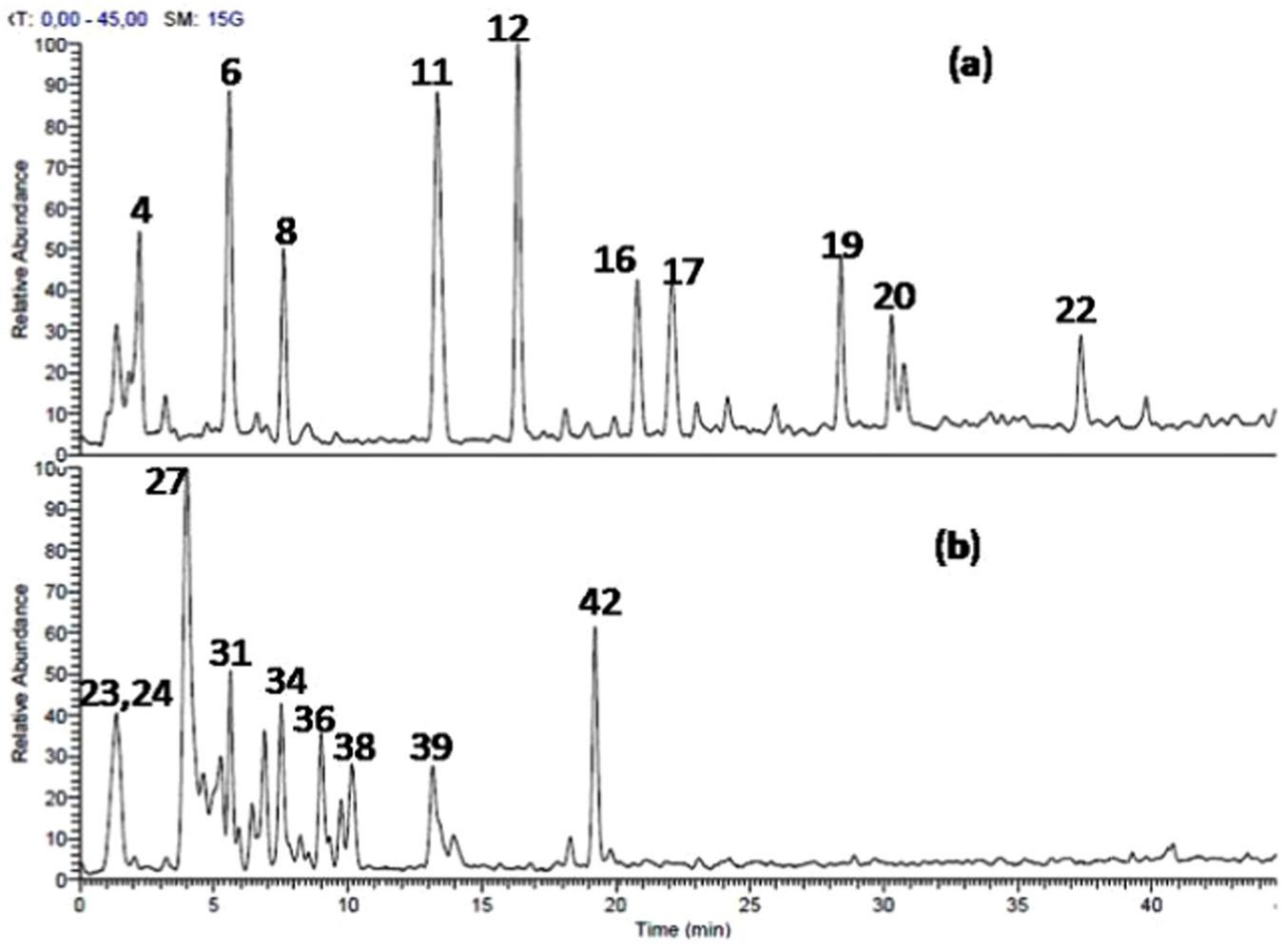
Total ion chromatogram of methanol bark extracts of (**a**) *L*. *stuhlmannii* (22 compounds, mainly tannins). (**b**) *L*. *humilis* (20 secondary metabolites, mainly sulphated tannins).

The methanol extracts obtained from the bark of *L*. *stuhlmannii* and *L*. *humilis* were analyzed by HPLC-PDA-ESI-MS/MS. A total of 22 and 20 secondary metabolites were tentatively identified in *L*. *stuhlmannii* and *L*. *humilis* bark, respectively. *L*. *stuhlmannii* extract is rich in condensed tannins, whereas sulphated condensed tannins and phenolic acids dominated in *L*. *humllis* (Table 1 and Fig. 1).

**Table 1:** Tentative identification of secondary metabolites in  methanol extracts  from bark of *L*. *stuhlmannii* and *L*. *humilis* by HPLC-MS/MS.

No.	Tentatively identified compounds	t_R_ (min.)	[M-H]^−^	MS/MS fragment
**Bark extract L. stuhlmannii**				
1	Malic acid^a^	1.32	133	115
2	Quinic acid^b^	1.32	191	
3	Caffeoylquinic acid^c^	2.04	353	173, 179, 191
4	Feruloylquinic acid	2.21	367	191, 193
5	(epi)-Catechin-(epi)-gallocatechin	4.71	593	245, 289, 425
6	(epi)-Catechin-(epi)-catechin^b^	5.59	577	289, 407, 425
7	Procyanidin dimer rhamnoside	6.62	723	407, 553, 571
8	(epi)-Catechin^b^	7.61	289	179, 205, 245
9	(epi)-Catechin-(epi)-catechin-(epi)-catechin^b^	8.41	865	287, 425, 577, 695
10	(epi)-Catechin-(epi)-afzelechin	9.54	561	289, 407, 425, 435
11	(epi)-Catechin-(epi)-catechin^b^	13.28	577	289, 407, 425, 451
12	(epi)-Catechin^b^	16.33	289	137, 179, 205, 245
13	Procyanidin dimer mono gallate^b^	18.08	729	407, 425, 559, 577
14	Procyanidin dimer mono gallate^b^	18.90	729	289, 425, 559, 577
15	(epi)-Catechin-(epi)-afzelechin^b^	19.94	561	289, 425, 435, 543
16	(epi)-Catechin-(epi)-catechin-(epi)-catechin^b^	20.85	865	287, 425, 577, 695
17	Procyanidin dimer mono gallate^b^	22.09	729	407, 425, 559, 577
18	Ligustroside	25.92	523	361
19	(epi)-Catechin gallate^b^	28.38	441	169, 271, 289
20	(epi)-Catechin-(epi)-catechin^b^	30.29	577	289, 407, 425, 451
21	(epi)-Catechin-A-(epi)-catechin	30.89	575	289, 423, 539
22	Ellagic acid coumaroyl-hexoside	37.39	609	301, 257
**Bark extract L. humilis**				
23	Malic acid^b^	1.31	133	115
24	Quinic acid^b^	1.40	191	127, 173
25	Gallic acid glucoside	2.03	331	169
26	(epi)Gallocatechin	3.23	305	179, 287
27	(epi)Gallocatechin 5-O-methyl 7-O-sulphate	3.87	399	193
28	(epi)Catechin-(epi)catechin	4.6	577	245, 289, 425, 559
29	(epi)-Gallocatechin-(epi)-gallocatechin gallate^b^	4.92	761	305, 423, 593, 609
30	Flavan 3-,4-,5- trihydroxy5-O-methyl 7-O-sulphate	5.28	383	125, 177, 285, 303
31	Syringic acid sulphate	5.65	277	197
32	(epi)Catechin^b^	6.41	289	179, 205, 245
33	(epi)Catechin 5-O-ethyl 7-O-sulphate-3-O-hexoside	6.87	559	317, 397, 479
34	(epi)Catechin 5-O-ethyl 7-O-sulphate	7.51	397	151, 289, 317
35	(epi)Gallocatechin gallate-(epi)catechin	8.22	745	287, 423, 457, 593
36	(epi)-Catechin gallate- (epi)gallocatechin	9.07	745	289, 407, 441, 593
37	(epi)Catechin^b^	9.77	289	179, 205, 245
38	(epi)Gallocatechin gallate^b^	10.13	457	169, 287, 331
39	Procyanidin dimer mono gallate^b^	13.13	729	289, 407, 441, 559
40	(epi)Gallocatechin gallate 5-O-ethyl 7-O- sulphate	13.92	551	193, 301, 319, 471
41	(epi)Catechin gallate-(epi)catechin gallate	18.30	881	407, 441, 559, 729
42	(epi)Catechin gallate^b^	19.22	441	169, 289

^a^Identification was based on^21^.

^b^Identification was based on^18^.

^c^Identification was based on^22^.

**Figure 2:**
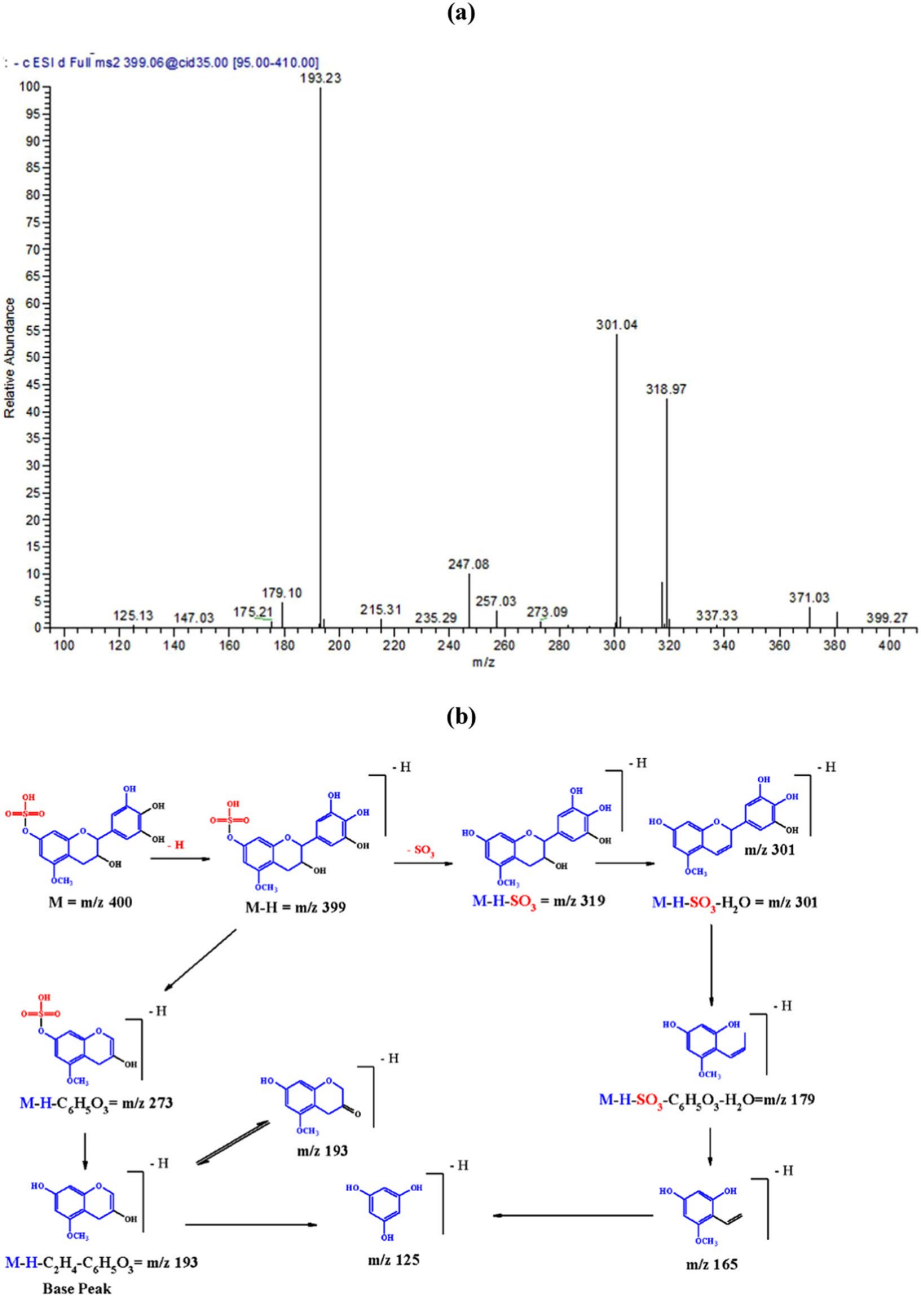
(**a**) MS^2^ of compound (**27**), (epi)gallocatechin 5-*O*-methyl 7-*O*-sulphate at [M – H]^−^ at *m/z* 399. (**b**) A proposed fragmentation pattern.

**Figure 3:**
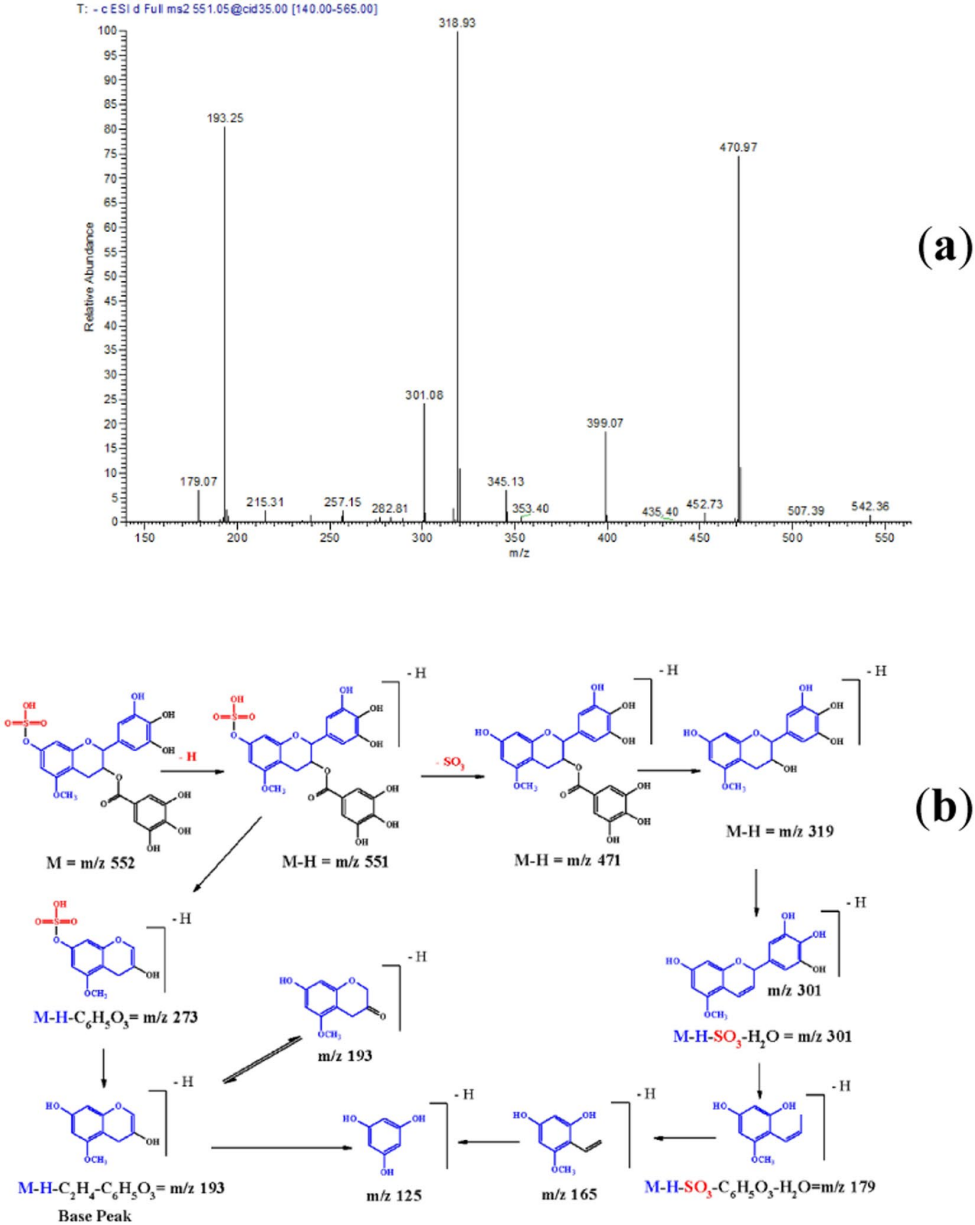
(**a**) MS^2^ of compound (**40**), (epi)gallocatechin gallate 5-*O*-methyl 7 sulphate at [M – H]^−^ at *m/z* 551. **(b)** A proposed fragmentation pattern.

In the *L*. *humilis* extract, compound **27**, (λ_max_ = 278), demonstrated a [M – H]^−^ at *m/z* 399 and main fragments at *m/z* 319 [M – H – 80, sulphate moiety], *m/z* 301[M – H – 80 – 18, sulphate and water moieties], and *m/z* 193 [M – H - C_2_H_4_ - C_6_H_5_O_3_]; it was tentatively characterized as (epi)gallocatechin 5-*O*-methyl 7-*O*-sulphate. Representative spectra and proposed fragmentation pattern are shown in Fig. 2(a,b). Also, compound **40** showed a [M – H]^−^ at *m/z* 551 and main daughter ions at *m/z* 471 [M – H – 80, sulphate moiety], 319 [M – H – 80–152, loss of sulphate and gallate moieties], 301, and 193, it was tentatively assigned to (epi)gallocatechin gallate 5-*O*-methyl 7-*O*- sulphate, (Fig. 3a,b).

**Figure 4:**
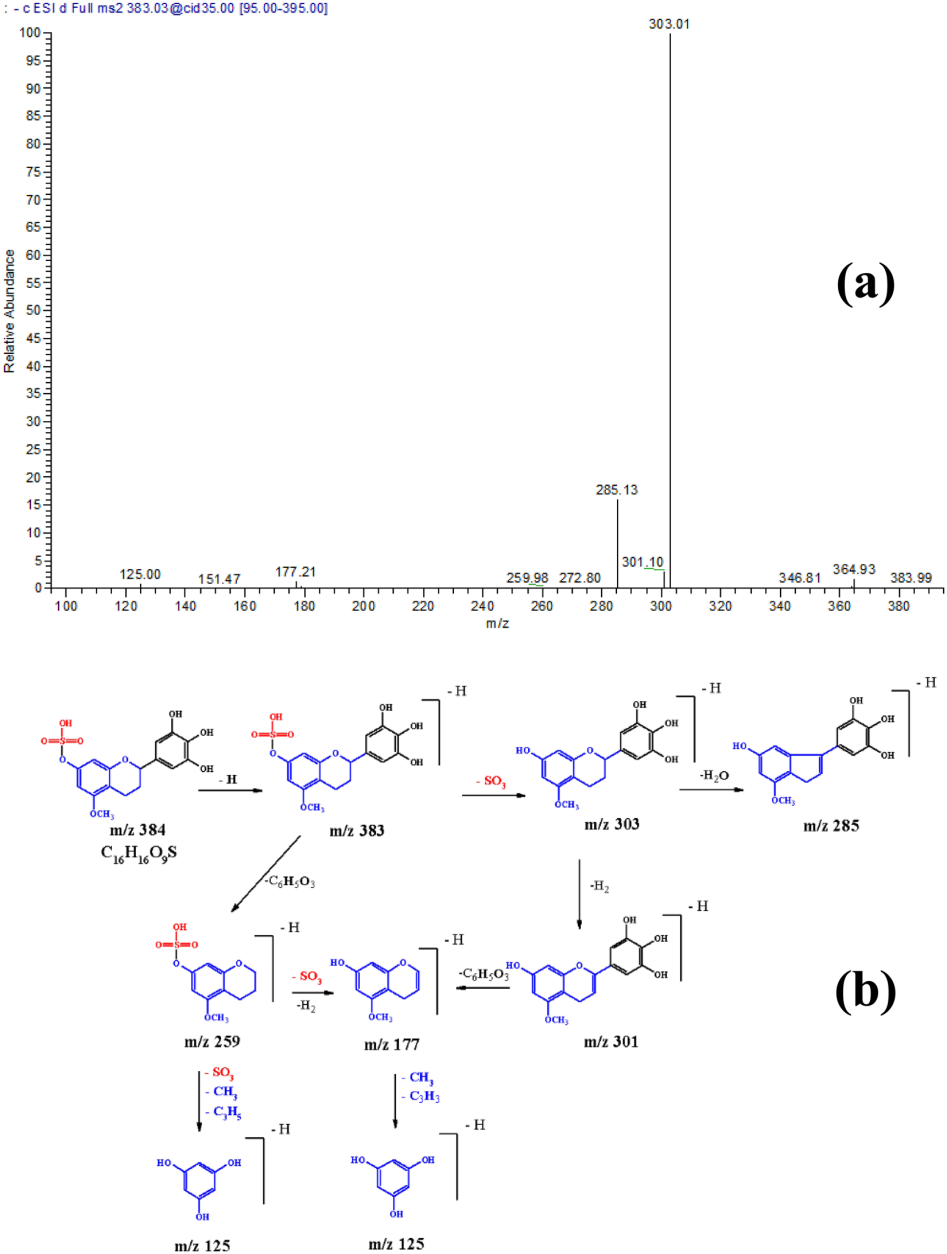
(**a)** MS^2^ of compound (**30**), flavan 3-,4-,5-trihydroxy 5-*O* methyl 7-*O*-sulphate at [M – H]^−^ at *m/z* 383. **(b)** A proposed fragmentation pattern.

Compound **30** exhibited a [M – H]^−^at *m/z* 383 and a main daughter ion at *m/z* 303 [M – H – 80, sulphate moiety]; it was tentatively identified as flavan 3-,4-,5-trihydroxy 5-*O* methyl 7 sulphate, (Fig. 4a,b**)**. Compound **31** exhibited a [M – H]^−^ at *m/z* 277 and a molecular ion peak at *m/z* 197 [M – H – 80]; it was assigned to syringic acid sulphate.

**Figure 5:**
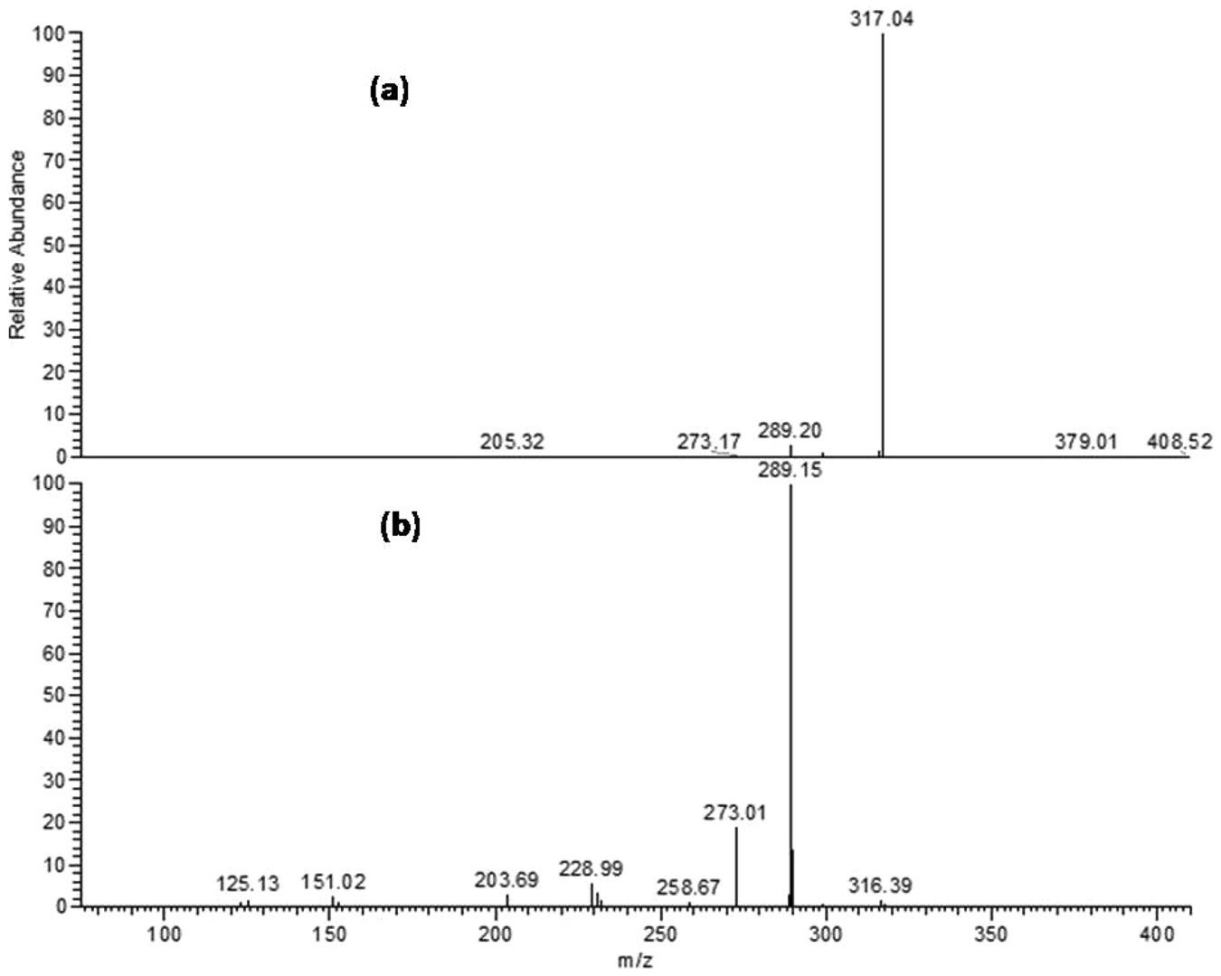
Negative ion ESI-MS/MS spectra of (epi)catechin-5-*O*-ethyl, 7-*O*-sulphate (**a**) MS^2^ of [M − H]^−^*m*/*z* 397. (**b**) MS^3^ of main daughter ion at *m*/*z* 317.

**Figure 6:**
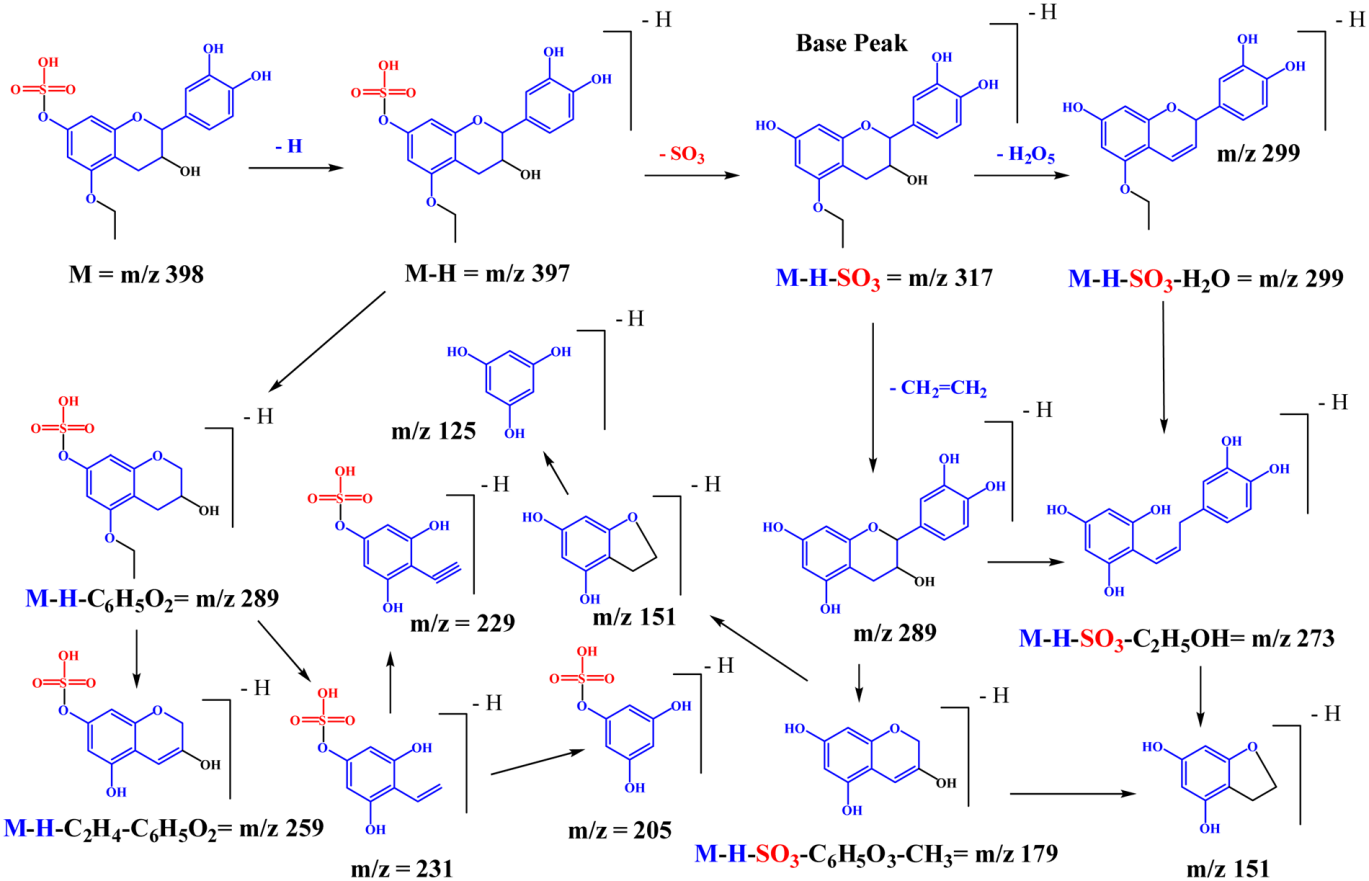
A proposed fragmentation pattern of (epi)catechin-5-*O*-ethyl 7-*O*-sulphate at [M − H]^−^*m*/*z* 397.

The peak at retention time 7.51 min (compound **34**) showed a [M – H]^−^ at *m/z* 397 and a main fragment ion at *m/z* 317. The latter exhibited a main fragment ion at *m/z* 289 in MS^3^. From the fragmentation pattern, we were not able to confirm the position of the ethyl group substituent, position 5 or position 8 on the flavan nucleus. After comparing its UV spectra with those of compound **27**; it was identified as (epi)catechin-5-*O*-ethyl 7-*O*-sulphate. According to Woodward’s rules, the compound would have had a higher λ_max_ than the current reported value if the ethyl group had been in position 8. A representative mass spectra and proposed fragmentation pattern are shown in Figs 5 and 6.

**Figure 7:**
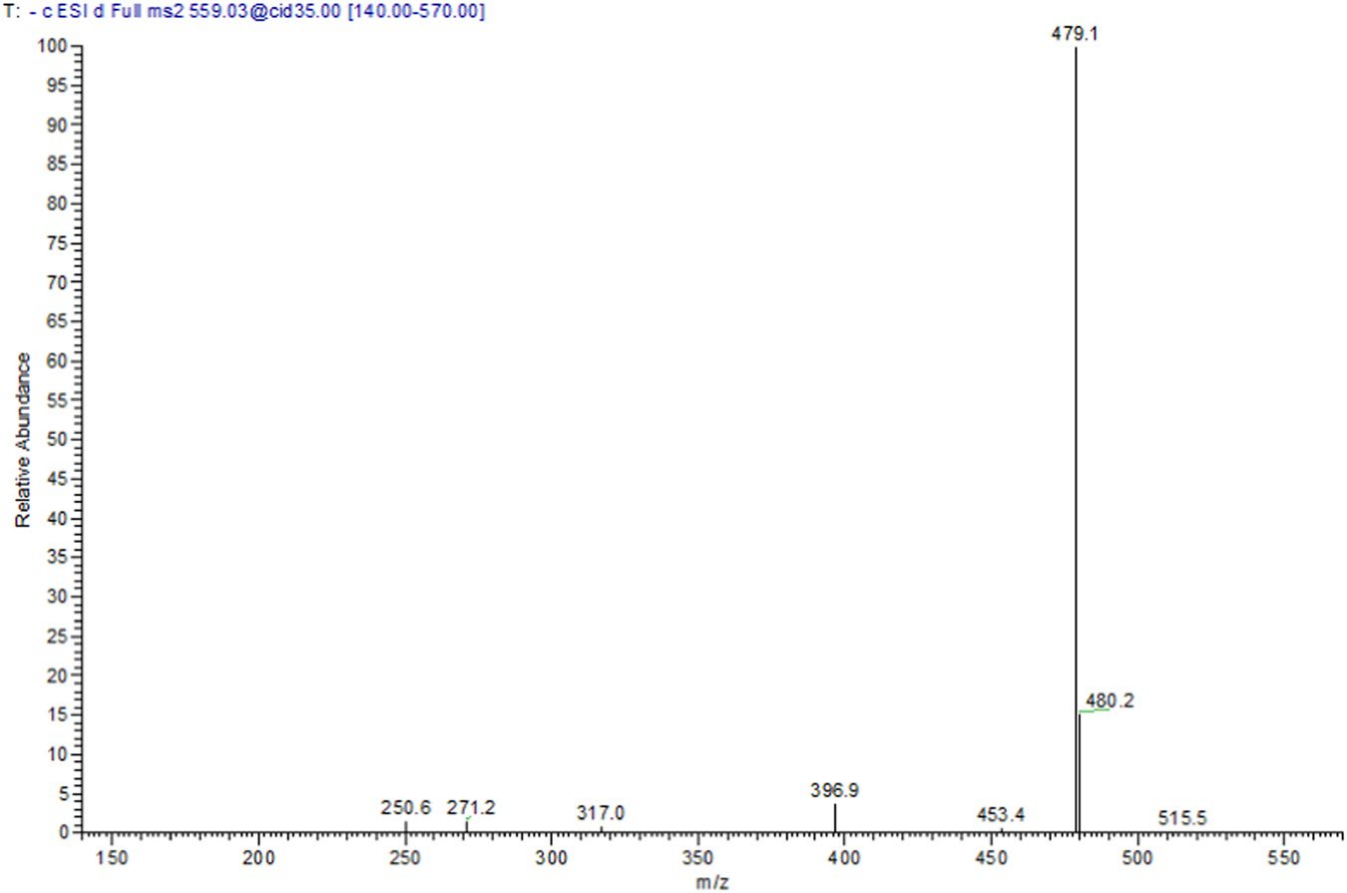
Negative ion ESI-MS/MS spectra of (epi)catechin-5-*O*-ethyl 7-*O*-sulphate 3-*O*-hexoside at [M − H]^−^*m*/*z* 559.

Peak **33** (retention time 6.87 min) has demonstrated a [M – H]^−^ at *m/z* 559 and fragment ions at *m/z* 479 [M – H – 80, sulphate moiety], 397 [M – H – 162, hexose moiety], and 317 [M – H – 80–162] was tentatively assigned to (epi)catechin-5-*O*-ethyl 7-*O*-sulphate 3-*O*-hexoside, Fig. 7.

Both (epi)gallocatechin gallate-(epi)catechin (**35**) and (epi)catechin gallate-(epi)catechin gallate (**41**) were detected only in *L*. *humilis* at [M – H]^−^ *m/z* 745, and 881, respectively, while caffeoylquinic acid (**3**) and feruloylquinic acid (**4**) were only detected in *L*. *stuhlmannii* at [M – H]^−^ *m/z* 353, and 367 with a molecular ion peak at *m/z* 191, respectively^22^. Both extracts were found to contain (epi)catechin monomers, dimers and trimers^17^.

### Biological activities

#### Antioxidant and hepatoprotective activities

Catechins are renowned for their powerful potential to scavenge various free radicals such as hydroxyl, peroxyl, superoxide, and other radicals. Antioxidant activity of catechins is mediated through different mechanisms. They are able to transfer an electron to bind a reactive radical, while they change to the more stable and less reactive phenoxyl radical. They are also able to chelate Cu^2+^ and Fe^3+^ ions, thus limiting free radicals generation. Indirectly, catechins exert an antioxidant effect *via* increasing the level of endogenous antioxidants such as glutathione reductase, catalase, and superoxide dismutase. Moreover, catechins are reported to have an inhibitory effect on xanthine oxidase that catalyzes the metabolism of purines into uric acid and reactive oxygen species. A direct relationship between the number of the hydroxyl groups a catechin possess and the exerted antioxidant potential has been demonstrated^17,18^.

Here, the antioxidant potential of the methanol extract of bark from *L*. *humilis* and *L*. *stuhlmannii* was investigated using two assays, namely DPPH and FRAP. The total phenolic content (TPC) was determined using Folin Ciocalteu assay and expressed as mg gallic acid (GAE)/g extract. Both extracts showed substantial activities in all assays, data are shown in Table 2. Such promising scavenging activities were reported from other *Lannea* species such as *L*. *alata*^4^, other bark-tannins rich extracts from *Albizia harveyi*, *Senna singueana*, *Turraea fischeri*, and proanthocyanidin rich root extracts from *Cassia abbreviata* and *Ximenia americana* var. *caffra*^17,18,21,23,24^.

**Table 2:** Total phenolic content (TPC), DPPH and FRAP assays of *L*. *humilis* and *L*. *stuhlmannii* bark.

Bark extract	TPC (mg GAE/g extract)	DPPH	FRAP
		(EC_50_ µg/mL)	(mM FeSO_4_ equivalent/mg sample)
*L*. *humilis*	275	9.3	19.77
*L*. *stuhlmannii*	336	5.6	18.32
Ascorbic acid	—	2.92	—
Quercetin	—	—	24.04

**Figure 8:**
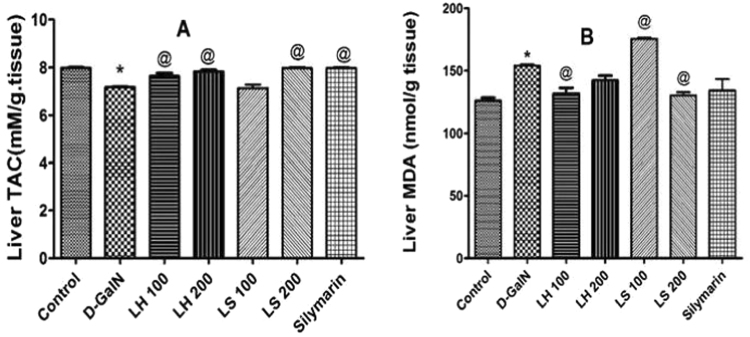
Antioxidant activity *in vivo* of oral administration of *L*. *humilis* (LH) and *L*. *stuhlmannii* (LS) in a dose of 100 mg/kg and 200 mg/kg and silymarin in a dose of 100 mg/kg for 3 days against the oxidative stress caused by D-galactosamine (D-GalN, 800 mg/kg, ip). (**A**) Total antioxidant capacity (TAC) in control and treated rats. (**B**) Liver malondialdehyde (MDA) contents in control and treated rats. Each bar represents the mean ± standard error (*n* = 5). *p < 0.05 versus control. ^@^*p* < 0.05 versus vehicle-treated D-GalN group.

In another set of experiments, the *in vivo* antioxidant potential was examined in rats treated with toxic D-galactosamine. We determined the total antioxidant capacity (TAC) and lipid peroxidation product, MDA in liver tissues as markers for oxidative stress. Except for the low dose level of *L*. *stuhlmannii*, both extracts were able to counteract the D-galactosamine-intoxication and increased the TAC of liver tissues (Fig. 8A). However, the low dose levels of *L*. *humilis* and *L*. *stuhlmannii (*100 mg/kg b.w.) were able to attenuate the MDA increase (Fig. 8B).

### Extract effects on markers of liver dysfunction and histopathological changes

**Figure 9:**
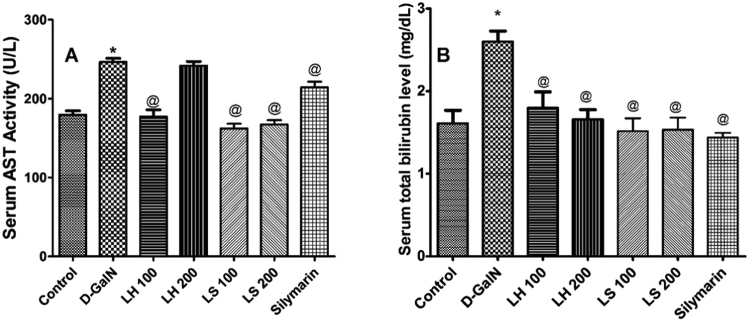
Hepatoprotective action of oral administration of *L*. *humilis* (LH) and *L*. *stuhlmannii* (LS) in a dose of 100 mg/kg and 200 mg/kg and silymarin in a dose of 100 mg/kg for 3 days in rats after D-galactosamine (D-GalN, 800 mg/kg, ip) induced acute liver injury. (**A**) Serum enzyme profile of AST in control and treated rats. (**B**) Serum total bilirubin level in control and treated rats. Each bar represents the mean ± standard error (*n* = 5). *p < 0.05 versus control. ^@^*p* < 0.05 versus vehicle-treated D-GalN group.

To test whether the two studied *Lannea* species have hepatoprotective activities *in vivo*, the biochemical and histopathological changes were evaluated after a liver cell injury had been induced by single dose injection of D-galactosamine (D-GalN, 800 mg/kg, i.p.). All treatments reduced AST and total bilirubin levels (*p* < 0.05), except the high dose of *L*. *humilis* which failed to decrease serum AST activity (*p* > 0.05), Fig. 9.

**Figure 10:**
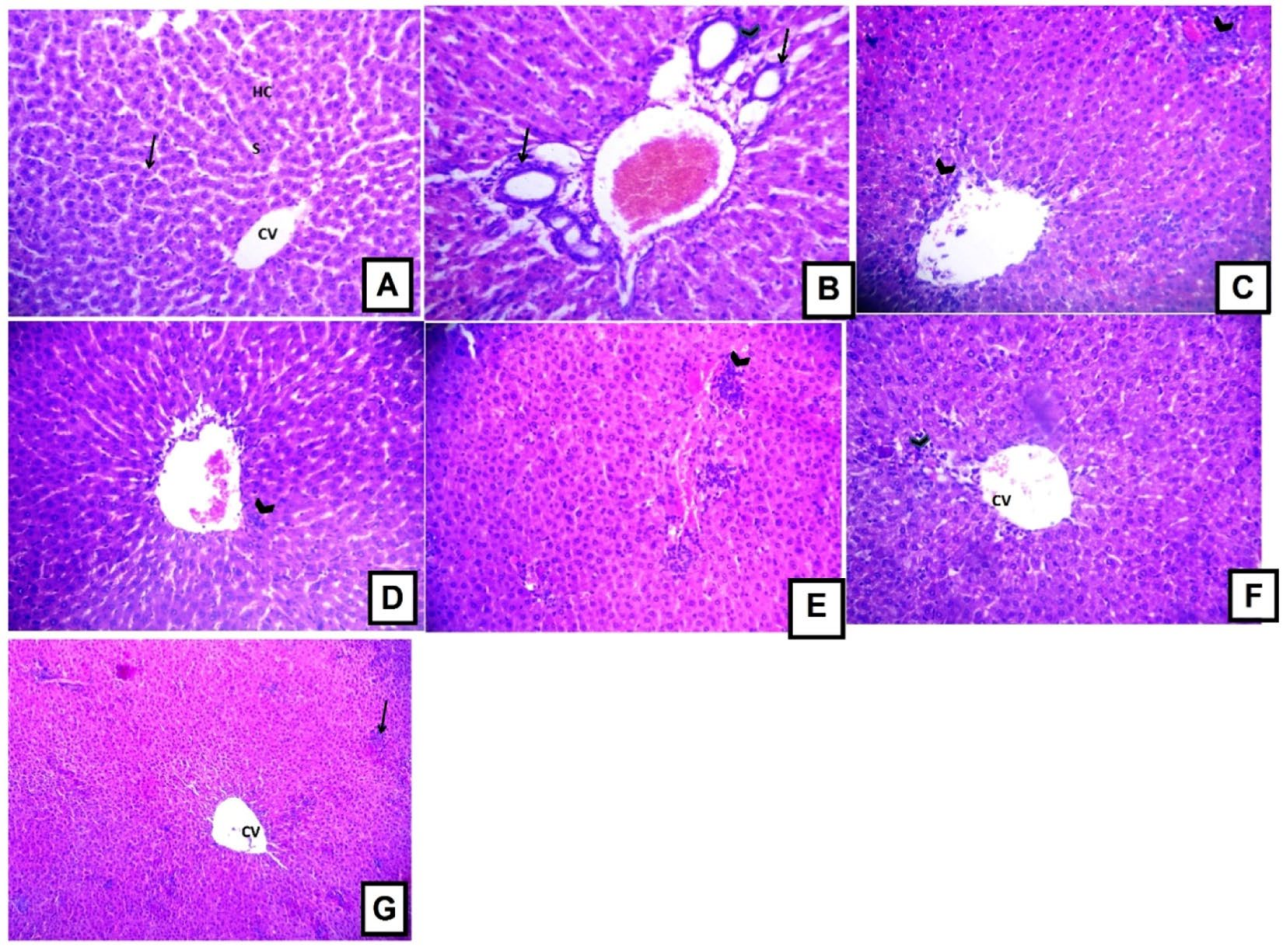
Light photomicrographs of the liver of rats treated with vehicle (0.9% saline), 200×. (**A**) Normal control. (**B**) The heptotoxic D-GaIN (800 mg/kg b.w.) (**C**,**D**) *L*. *humilis* (100 and 200 mg/kg, b.w.), (**E**,**F**) *L*. *stuhlmannii* (100 and 200 mg/kg, b.w.) (**G**) The hepatoprotective control silymarin (100 mg/kg, b.w.).

To confirm the *in vivo* antioxidant activities and the observed biochemical changes, a histopathological evaluation was performed. It is obvious from Fig. 10B that D-GalN has induced a mononuclear cellular infiltration indicating the development of inflammation in liver tissues (arrow head) in the portal area and congested blood vessel (BV). Together with the observed hyperplasia of bile ducts (arrow), these changes provide evidence for the successful establishment of animal liver injury model. In general, these deleterious changes were significantly attenuated by *L*. *humilis* and *L*. *stuhlmannii* treatment. However, a dose of 100 mg/kg *L*. *humilis* has shown little improvement and some mononuclear cellular infiltration (arrow) was spotted in the portal tract area and additionally a congested blood vessel (BV) was detected (Fig. 10C).

A higher dose of *L*. *humilis* (200 mg/kg) showed marked improvement indicated by little mononuclear cellular infiltration (arrow) and central vein (CV) with minimum congestion, (Fig. 10D**)**. The low dose level (100 mg/kg) of *L*. *stuhlmannii* had little improvement as seen in Fig. 10E, where mononuclear cellular infiltration (arrow head) was clearly noticed in the portal area accompanied with bile duct hyperplasia (arrow). Figure 10F revealed that the high dose level (200 mg/kg) of *L*. *stuhlmannii* was able to exert some improvements where mononuclear cellular infiltration was observed only in the portal tract area (arrow head) and less congested blood vessel (BV) was also observed. Figure 10G showed that silymarin pretreatment (a lignin with known hepatoprotective properties) caused partial improvement.

### Extract effects on anti-apoptotic marker Bcl-2

**Figure 11:**
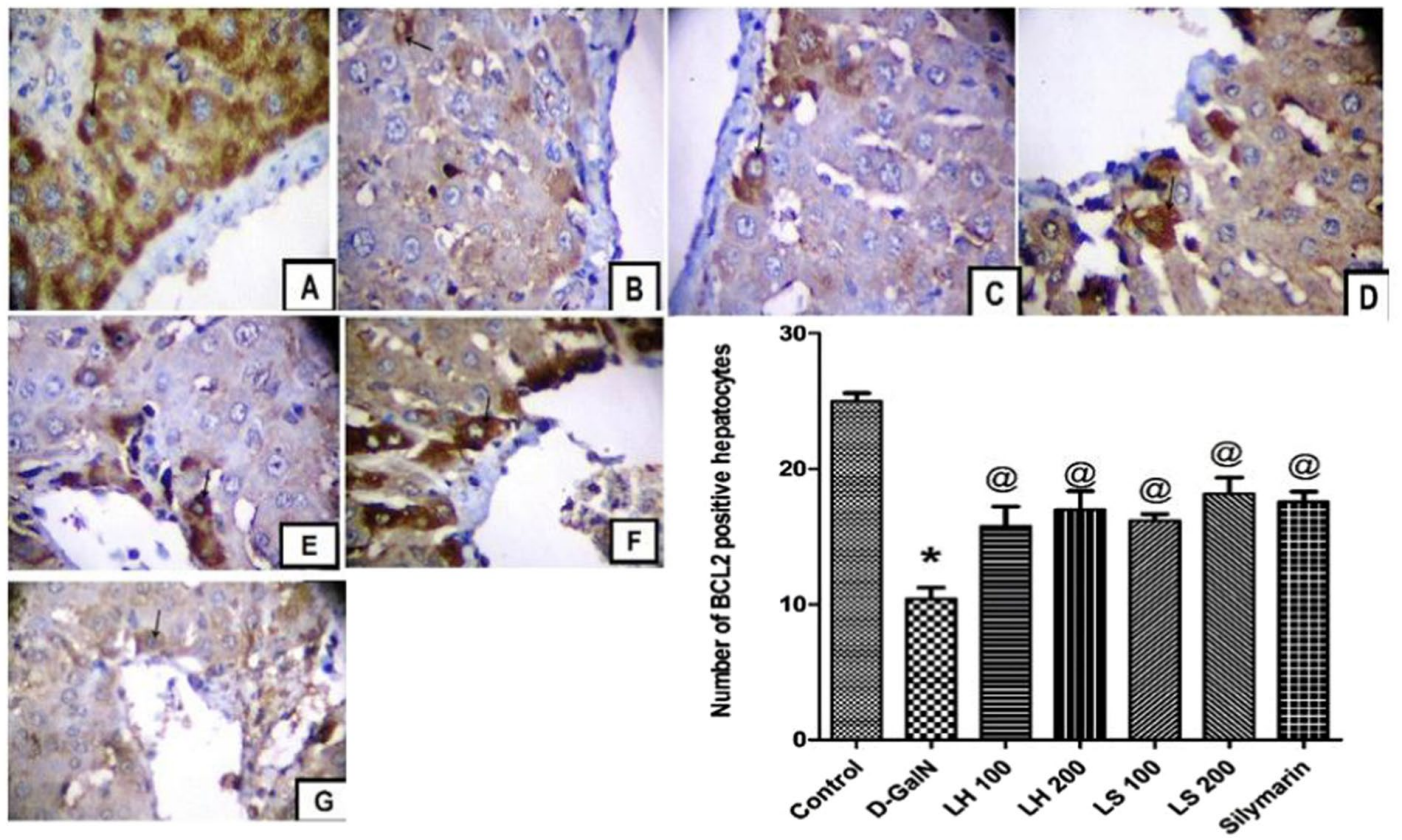
Immunohistochemical determination of Bcl-2 in paraffin-embedded liver tissues stained with Avidin-biotin peroxidase stain with haematoxylin counter stain, (**A**) Liver of rats treated with vehicle (0.9% saline). (**B**) Rats injected a single dose of D-GaIN (800 mg/kg, b.w.); (**C** and **D**) rats treated with two doses of *L*. *humilis* for 3 days (100 and 200 mg/kg, b.w.). (**E** and **F**) Rats treated with two doses of *L*. *stuhlmannii* for 3 days (100 and 200 mg/kg, b.w.). (**G**) The positive control silymarin (100 mg/kg, b.w.), magnification, 400×. The Bar graph represents mean ± standard error of the mean of the number of Bcl-2 positive hepatocytes (*n* = 6). *p < 0.05 versus control, ^@^*p* < 0.05 versus vehicle-treated D-GalN group.

When cellular antioxidants are depleted and ROS accumulation reaches a critical threshold, mitochondrial damage occurs. This process leads to the release of cytochrome c (Cyt-c) from the mitochondria, which in turn activates the apoptotic pathway^25^. Several proteins can inhibit or decrease apoptosis, among them the anti-apoptotic members of Bcl-2 family proteins^26^. To clarify the mechanism that the extracts attenuated hepatotoxicity induced by D-GalN, we stained liver sections with avidin-biotin peroxidase stain with haematoxylin counter stain for Bcl-2, anti-apoptotic marker, and determined the percentage of positively stained hepatic cells. A significant increase in the number of Bcl-2 positive hepatocytes was observed in rats treated with all dose levels of the extracts (Fig. 11C–F), suggesting that both *Lannea* species protected hepatocyte from apoptotic cell death. The extracts produced similar effect to silymarin (Fig. 11G).

### Molecular modelling study

The family of B-cell lymphoma-2 (Bcl-2) proteins is known for regulating programmed cell death through mediating mitochondrial or intrinsic initiated apoptosis. The Bcl-2 proteins are either pro-survival (anti-apoptotic) such as Bcl-XL, Bcl-2, Bcl-B, and Mcl-1 proteins or anti-survival (pro-apoptotic) proteins including Bim, Bad, Bak, Bax, and others^27^.

The molecular structures of the majority of these proteins have been resolved. The anti-apoptotic members have been found to share one or even more of the crucial BH domains namely BH1, BH2, BH3, and BH4. All the pro-apoptotic proteins, however, contain the BH3 domain that is very essential for cell killing activity^28^.

**Figure 12:**
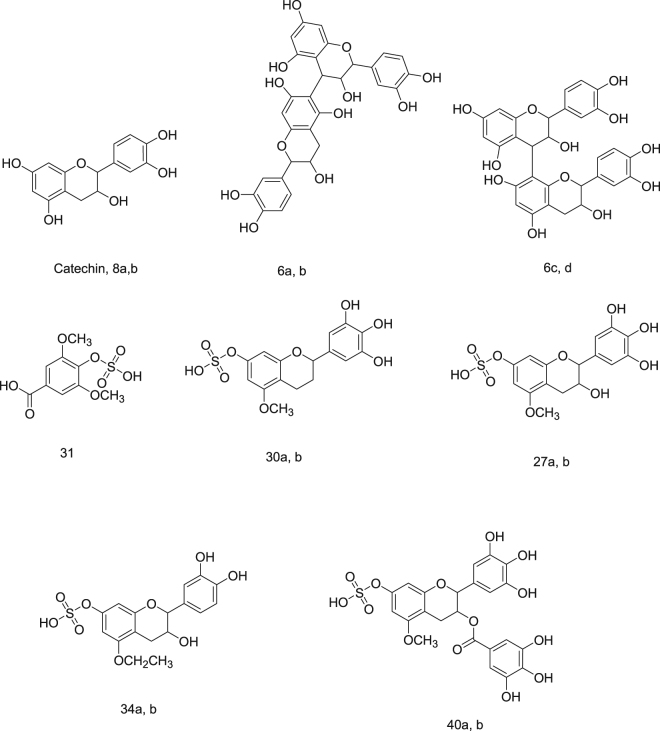
Compounds docked to the Bcl-2: Bim (BH3) interface. Compounds are numbered according to Table 1.

In order to get an insight how the plant extracts were able to protect against hepatic cells death, we decided to dock some of the identified compounds to the crystal structure of the interaction surface of Bcl-2:Bim (BH3) complex, pdb code: 4b4s, using molecular operating environment (MOE), 2013.08; Chemical Computing Group Inc., Montreal, QC, Canada, H3A 2R7, 2016 according to our previously applied protocol^24^. The binding site in the Bcl-2: Bim (BH3) crystal structure as well as the amino acid residues crucial for the efficient binding between the two proteins are well established, thus docking the plant major chemical components to this site would be beneficial to understand how they are able to hinder the Bcl-2: Bim complex formation and thus blocking apoptosis. The docked compounds are shown in Fig. 12 and docking results are summarized in Table 3.

**Table 3:** Scoring function of docking poses and the interactions of the docked compounds with amino acid residues on Bcl-2: Bim (BH3) interface.

	Stereo-center(s) configuration	Scoring function	Amino acid residues interactions
Catechin	2R, 3S	−11.98	Tyr 72 (H-bonding) Arg 44 (H-bonding, Hydrophobic) Ser 40 (Hydrophobic) Phe 163 (Hydrophobic)
8a	2S, 3S	−15.17	Ser 40 (H-bonding) Phe 159 (H-bonding) Tyr 73 (Hydrophobic) Phe 163 (Hydrophobic)
8b	2R, 3R	−12.34	Gln 47 (H-bonding) Ser 40 (Hydrophobic) Arg 44 (Hydrophobic)
6a	All S	−15.49	Tyr 73 (H-bonding) Tyr 72 (Hydrophobic) Arg 160 (Hydrophobic)
6b	All R	−14.62	Phe 159 (H-bonding through solvent) Thr 161 (H-bonding through solvent) Arg 160 (Hydrophobic) Ala 36 (Hydrophobic) Arg 44 (Arene-Cation)
6c	All S	− 15.27	Ser 40 (H-bonding) Arg 44 (H-bonding through solvent) Phe 163 (H-bonding through solvent)
6d	All R	−14.22	Gln 47 (H-bonding) Phe 159 (H-bonding through solvent) Thr 161 (H-bonding through solvent)
31	----	−13.08	Tyr 73 (H-bonding) Ser 40 (H-bonding) Phe 159 (H-bonding through solvent) Thr 161 (H-bonding through solvent) Arg 44 (Hydrophobic)
30a	2S	−13.46	Ser 40 (H-bonding) Tyr 72 (Hydrophobic) Arg 44 (Arene-Cation)
30b	2R	−12.34	Arg 44 (H-bonding, Arene-Cation) Phe 163 (Hydrophobic)
27a	2S, 3S	−14.50	Tyr 73 (H-bonding) Phe 159 (H-bonding through solvent) Thr 161 (H-bonding through solvent) Ser 40 (Hydrophobic) Arg 44 (Hydrophobic)
27b	2R, 3R	−14.09	Ser 40 (H-bonding) Arg 44 (H-bonding) Tyr 73 (H-bonding) Phe 159 (H-bonding through solvent) Thr 161 (H-bonding through solvent)
34a	2S, 3S	− 12.73	Arg 44 (H-bonding) Tyr 73 (H-bonding) Ser 40 (Hydrophobic)
34b	2R, 3R	−12.97	Arg 44 (H-bonding) Phe 159 (H-bonding through solvent) Thr 161 (H-bonding through solvent)
40a	2S, 3S	−14.15	Tyr 73 (H-bonding) Arg 44 (H-bonding and hydrophobic) Ser 40 (Hydrophobic)
40b	2R, 3R	−14.47	Tyr 73 (H-bonding) Phe 159 (H-bonding) Arg 160(H-bonding) Ser 40 (H-bonding) Phe 163 (Hydrophobic)

Despite the fact that the actual mechanism by which these proteins regulate apoptosis is still not clearly understood, it is well accepted now that this happens through heterodimerization of two members of pro- and anti-apoptotic agents into a protein complex that activates apoptotic pathways^29^. Resolving the loop residues in the Bcl-2:Bim (BH3) complex for instance, has shown that Bcl-2 surface has a hydrophobic cleft in which the hydrophobic face of the BH3 of Bim binds. In this regard, Bim (BH3) domain was shown to extend from Arg 53 to Ala 74 amino acid residues. Within this domain, the side chains of five hydrophobic residues are interacting with the hydrophobic cleft of Bcl-2 and conserved in all pro-apoptotic BH3 domains, namely Ile 58, Leu 62, Ile 65, Phe 69, and Tyr 73^30^. Additionally, some more amino acid residues were later reported to contribute favorably to the binding free energy of the complex such as Arg 64 and Glu 68. The residue Tyr 72 contributes to a lesser extent to the complex formation as its side chain is more solvent exposed^31^. Studies have shown that a single mutation in any of these residues such as Leu 62, for instance, could lead to a severe impairment in the binding of the two opposing proteins and hence blocking the apoptotic cell death^32,33^.

**Figure 13:**
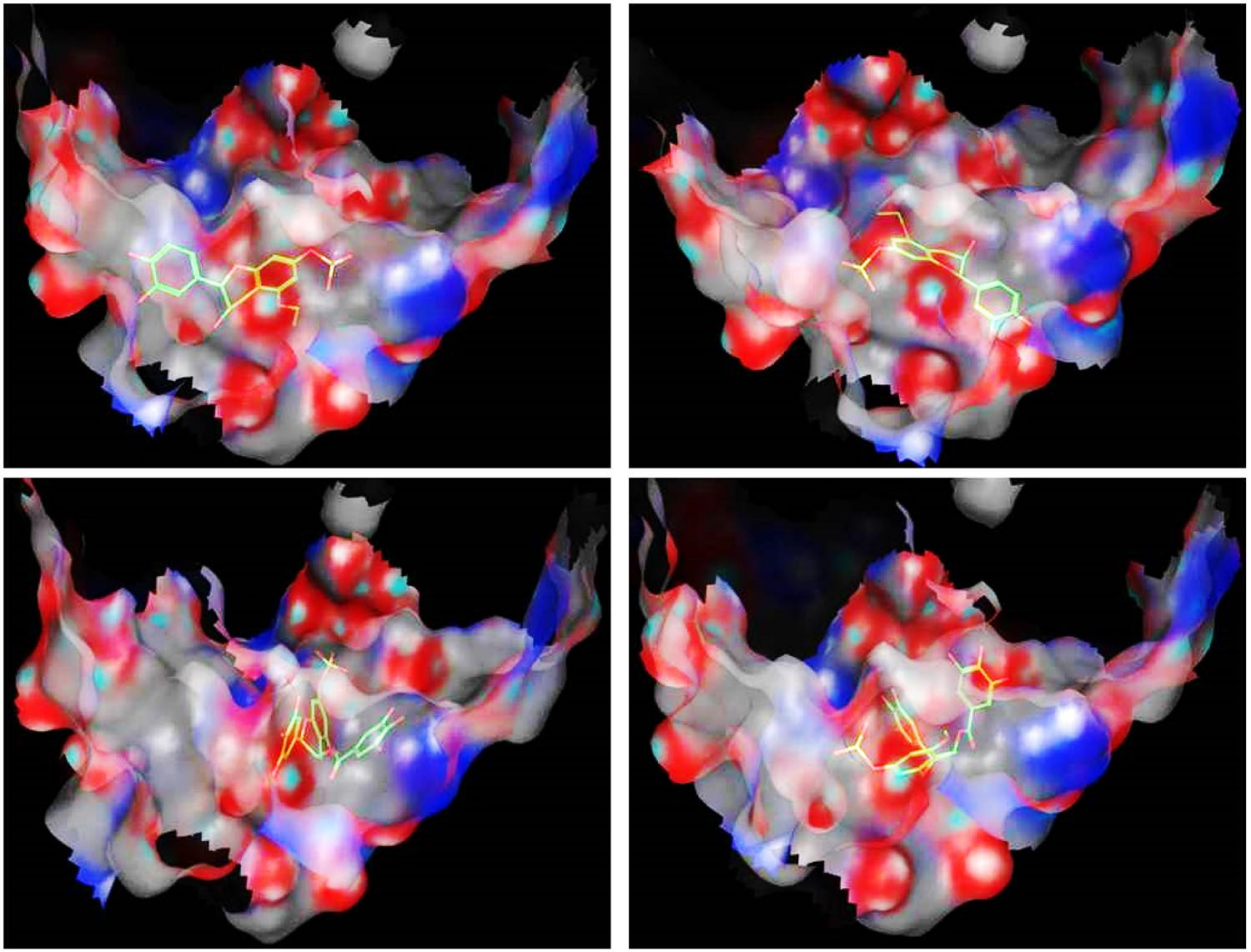
3D-docking poses of compounds 34a (top left), 34b (top right), 40a (bottom left), and 40b (bottom right) docked to Bcl-2: Bim (BH3) interface. Surface residues are coloured (grey hydrophobic, red acidic, blue basic, and cyan other).

**Figure 14:**
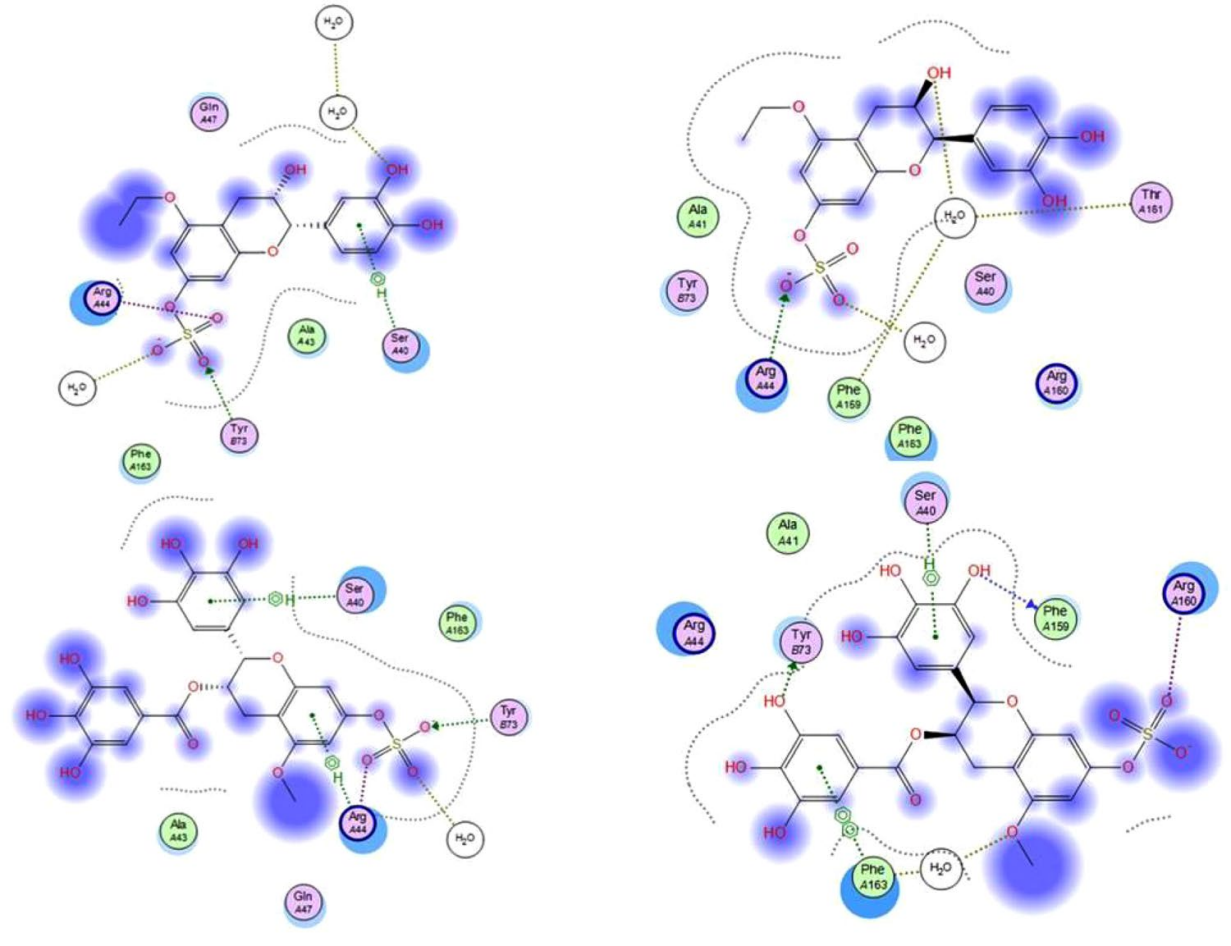
2D-interactions of compounds 34a (top left), 34b (top right), 40a (bottom left), and 40b (bottom right) with amino acid residues on Bcl-2: Bim (BH3) interface.

As shown in Table 3, the docked compounds were able to bind to the Bcl-2: Bim (BH3) interface with a scoring function of a range between −11.98 to −15.94 reflecting an appreciable binding free energy. Additionally, the compounds have afforded different polar and hydrophobic interactions to the amino acid residues in the hydrophobic face of Bim BH3 domain, (Table 3). Except for 6c, all other compounds with S-configuration of the stereo-center(s) were able to interact with the essential conserved Tyr 73 and/or Tyr 72 residues. However, compounds 27b and 40b having stereo-centers with R- configuration have also shown to interact with Tyr 73 residue. This interaction stabilized by different other polar and hydrophobic interactions could result in conformational changes in the protein structure leading to interference with dimerization of the two opposing proteins Bcl-2: Bim, thus hindering apoptosis. Figures 13 and 14 show the 3D poses and the 2D-interactions, respectively, of two selected compounds34a, 40a, and their enantiomers 34b, and 40b docked to the Bcl-2: Bim (BH3) interface.

In view of the immunohistochemical and molecular modeling studies, the anti-apoptotic effect of the extracts could be presumably attributed to their tannin content. This comes to agreement with other studies that have reported anti-apoptotic activity for proanthocyanidin rich extracts such as the bark extract of *Senna sengueana*^21^. Polyphenols are powerful biologically active natural products which can bind to several important proteins in cells by forming multiple hydrogen and ionic bonds that can modulate the 3D structure of proteins^34^. In summary, evidence is provided that *L*. *humilis* and *L*. *stuhlmanni* protect against D-GalN-induced liver injury. The hepatoprotection of the extracts is due to antioxidant and anti-apoptotic effects.

## Conclusions

The chemical profiling of the methanol extracts of *Lannea stuhlmannii* and *L*. *humilis* bark was analyzed by HPLC-PDA-ESI-MS/MS. A total of 42 secondary metabolites were identified in the investigated extracts. Noteworthy, this work provides the first comprehensive phytochemical study of *L*. *stuhlmannii* and *L*. *humilis* bark and confirms their traditional use. The extracts could protect rat liver from intoxication with D galactosamine, apparently through antioxidant and increasing the antiapoptotic protein Bcl-2. Thus, *Lannea* is another candidate for the potential treatment of liver injury that needs further and deeper analyses.
